# The protective effect of dietary supplementation of *Salmonella-specific* bacteriophages in post-weaning piglets challenged with *Salmonella typhimurium*

**DOI:** 10.5455/javar.2021.h532

**Published:** 2021-09-20

**Authors:** Yong-Kwan Won, Sung-Jae Kim, Jeong-Hee Han

**Affiliations:** 1 Department of Veterinary Pathology, College of Veterinary Medicine and Institute of Veterinary Science, Kangwon National University, Chuncheon, Republic of Korea; 2 Department of Veterinary Microbiology, College of Veterinary Medicine and Research Institute for Veterinary Science, Seoul National University, Seoul, Republic of Korea; † These authors contributed equally to this work.

**Keywords:** Bacteriophage, dietary supplement, *S. typhimurium*, piglets, diarrhea, growth performance, intestine

## Abstract

**Objective:**

The efficacy of *Salmonella typhimurium-*specific bacteriophage STP-1 on *S.* *typhimurium* infection in weaning piglets was evaluated in this study.

**Material and Methods:**

Twenty-eight weaning piglets were randomly allocated to four groups (Group A: non-challenged/basal; Group B: non-challenged/+phage; Group C: challenged/basal; Group D: challenged/+phage) according to *S. typhimurium* infection or bacteriophage administration. The total experimental period (14 days) was subdivided in to non-challenged periods (phase I; day 1–7) and challenged periods (phase II; day 7–14) based on the challenging date (day 7). Each group was fed with basal feed or feed supplemented with bacteriophage STP-1 [1.0 × 10^9^ plaque-forming unit (PFU)/kg] during the whole period (day 1–14). Body weights (BW) were measured to evaluate growth performance. Clinical symptoms (rectal temperatures and fecal consistency) induced by *S. typhimurium* were regularly checked. Bacteria colonization levels in feces and intestinal tissue samples were measured using real-time polymerase chain reaction (PCR). After necropsy, small intestine samples (jejunum) were collected. Villus height and crypt depth (CD) were measured through histological examination with H&E staining.

**Results:**

The supplementation of bacteriophage significantly reduced bacterial colonization and intestine damage in the piglets infected with *S. typhimurium*. In the antigen concentrations of the feces and jejunum, Group C showed 5.8 ± 0.6, 5.7 ± 0.6, and 1.2 ± 2.0 log colony-forming unit (CFU)/ml on 1, 3, and 7 days post-inoculation (DPI) and 2.8 ± 1.3 log CFU/ml, whereas Group D showed 3.5 ± 1.7, 2.2 ± 2.1, and 0.3 ± 0.9 log CFU/ml on 1, 3, and 7 DPI and 5.1 ± 0.9 log CFU/ml. In the villous height, Groups C and D showed 266.3 ± 24.1 and 324.6 ± 18.0 μm, respectively. In the goblet cell density of villi and crypts, Group C showed 10.0 ± 1.8 and 16.0 ± 3.7, while Group D showed 15.0 ± 4.8 and 21.1 ± 5.4. Also, the supplementation of bacteriophage significantly improved the growth performance in the infected piglets. The average daily gains of Groups C and D were 91 ± 24 and 143 ± 23, respectively, during the period after inoculation with *S. typhimurium*.

**Conclusion:**

The dietary supplementation of the phage was effective for alleviating *S. typhimurium* infection in post-weaning piglets.

## Introduction

Bacteriophage was first discovered by Twort and d’Herelle in 1910 and was used as a preventive remedy for bacterial diseases. However, early bacteriophage studies were not approached systematically, and these studies have not been continued due to the rapid development of chemotherapy, including antibiotics. Recently, the emergence and increase in pathogenic bacteria resistant to antibiotics have become a big problem [1]; there is a growing interest in alternative antibiotic therapy to solve these problems. Even if a new antibiotic that acts on antibiotic-resistant bacteria is developed with a huge development cost, there is a possibility that a new antibiotic-resistant bacteria may appear; thus, a therapeutic strategy to replace the antibiotic is needed [2]. Among antibiotic alternative treatment strategies, bacteriophage therapy has been receiving attention and many positive results have been reported on bacterial treatment [3].

Recently, studies on bacteriophage therapy for livestock bacterial diseases have been actively carried out. Bacteriophage therapies have been reported for bacteria, like *Salmonella* spp., *Escherichia* *coli*, *Campylobacter* spp., *Streptococcus* spp., etc., which cause diseases in not only livestock but also human health [4–8].

*Salmonella typhimurium* is not only the most common pathogen causing food poisoning in humans but it is most often isolated from livestock, primarily pigs. *S. typhimurium* is very high susceptible between 6 and 12 weeks, and inapparent infection is usually shown in adult pigs. These bacterial pathogens cause high morbidity and mortality and cause various symptoms, including diarrhea, systemic symptoms, such as fever, jaundice, and sepsis, and death [9].

Therefore, this study was conducted to evaluate the protective effect of bacteriophage against *S. typhimurium* infection, one of the causative pathogens of porcine diarrhea, which causes a severe problem in the swine industry. For evaluating the bacteriophage therapy, feed supplemented with *S. typhimurium*-specific bacteriophage was used, and clinical symptoms, growth performance, detection of antigens in feces and organs, and histopathological changes in the intestine were investigated according to *S. typhimurium* infection and bacteriophage administration in weaning piglets.

## Materials and Methods

### Preparation of S. typhimurium-specific phage

*S. typhimurium-*specific phage STP-1 (CTCbio Inc., Korea), which is a member of the Myoviridae family, was recovered from pig manure collected from a sewage treatment plant of a commercial swine farm. The collected manure solution was centrifuged (4,000 rpm, 15 min, 4°C). Subsequently, the supernatant was filtrated with 0.45 μm. The filtrate was mixed with *S. typhimurium* in tryptic soy broth (TSB), and then, the mixture was incubated for 18 h at 37°C with shaking at 200 rpm. The culture medium was centrifuged (4,000 rpm, 15 min, 4°C) and the supernatant was filtrated with 0.45 μm. The filtrate was overlaid on a plate spread with *S. typhimurium*. After incubation for 18 h at 37°C, plaques by phage were selected. For single isolation of phage, the plaque assay procedure was repeated thrice. The selected phages were diluted in a modified SM buffer (0.2 M Tris, pH 7.5, containing 0.1 M NaCl, 1 mM MgSO_4_, and 0.01% gelatin) and stored at 4°C. For amplification of the phage, *S. typhimurium* was cultured in TSB at 37°C until reaching OD 0.8, and the phage was inoculated to the culture medium. After inoculation of the phage, additional incubation at 37°C was carried out for 5 h. The culture medium was centrifuged (4,000 rpm, 15 min, 4°C). Subsequently, the supernatant was filtrated with 0.2 μm. Finally, the filtrate was powdered in 1.0 × 10^9^ PFU/gm concentration through the spray drying procedure before being used as a dietary supplement.

### Preparation of S. typhimurium for the challenge

*S. typhimurium* CTC1110, which was isolated from a piglet showing diarrhea in June 2011, was provided from CTCbio. Preculture was carried out in 10 ml of TSB at 37°C overnight. Subsequently, the main culture was carried out in TSB at 37°C for 18 h with shaking at 200 rpm. The cultured bacterial solution was confirmed by *S. typhimurium-*specific PCR [10]. Bacterial concentration (CFU/ml) was measured with 10-fold serial dilution. The culture medium was stored at 4°C overnight until confirming the CFU. The next day it was adjusted to 5.0 × 10^8^ CFU/5 ml in PBS and used for the challenge.

### Experimental design

**Figure 1.**
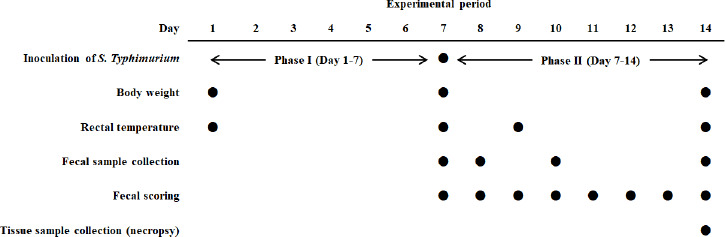
Schematic diagram of the experiment design.

The experiment protocol of this study was approved by the Institutional Animal Care and Use Committee of Kangwon National University. Twenty-eight 3w weaned piglets, which were *S. typhimurium*-negative as determined by *S. typhimurium-*specific PCR [10], were purchased from a commercial swine farm. The animals were allocated randomly in to four groups (Table 1) with seven animals per group/pen. Each of the pens was equipped with a nipple waterer and a feeder. The experiment was carried out as shown in Figure 1. Groups A and C were freely fed with an antimicrobial additive-free basal diet, the composition of which had been reported previously [11]. Groups B and D were freely fed with the same diet supplemented with 1.0 × 10^9^ PFU of the *S. typhimurium-*specific bacteriophage per kg during the whole experimental period. After 7 days of adaptation to the basal diet, the two challenged groups (Groups C and D) were challenged orally with 5.0 × 10^8^ CFU / 5 ml of *S. typhimurium*, and the two non-challenged groups (Groups A and B) were given the same volume of vehicle. BW was measured on days 1, 7, and 14 to evaluate the growth performance. After the challenge with *S. typhimurium*, the fecal consistency of each animal was daily scored according to a 4-notch scale as described previously [11]: 0 = normal; 1 = soft feces; 2 = mild diarrhea; 3 = severe diarrhea. Rectal temperatures were measured on days 1, 7, 9, and 14. Fecal samples were obtained from the rectum on days 7, 8, 10, and 14 to measure the shedding of *S. typhimurium*. 

**Table 1.** Experimental groups.

Group	No. of heads	Administration of bacteriophage (PFU/gm)	Inoculation of bacteria (CFU/ml)
A	7	-	-
B	7	1.0 × 10^9^	-
C	7	-	1.0 × 10^8^
D	7	1.0 × 10^9^	1.0 × 10^8^

### Necropsy

The piglets were euthanized by electric stunning at the end of the experiment. Subsequently, the middle part of the jejunum was removed from each piglet. Cross-sectional segments of the jejunum were obtained to measure *S. typhimurium* concentration by *S. typhimurium*-specific real-time PCR [10]. The histomorphological examination of their mucosae was carried out as per the method reported previously [11].

### Quantification of S. typhimurium in feces and small intestines

**Figure 2.**
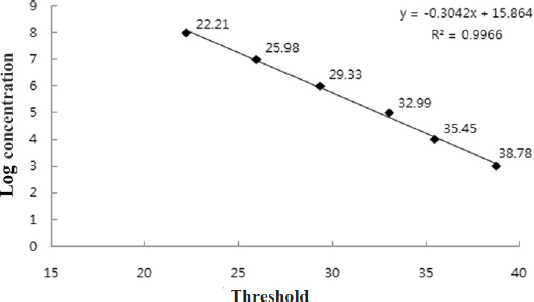
Standard curve of the real-time PCR for *S. typhimurium.* The results of the real-time PCR were determined using a decimal dilution of *S. typhimurium* DNA. The threshold values (CT) were plotted against the corresponding bacterial cell number (log_10_ CFU/ml).

The concentration of *S. typhimurium* in the feces and jejunum tissues was determined by real-time PCR targeting *S. typhimurium fliC* gene [10]. Briefly, genomic DNA was extracted from 50 mg of feces and tissues using QIAamp DNA Mini Kit (Qiagen, Germany). The PCR mixture contained 2 μl of DNA template, 1 μl (10 pmole/ul) of forward primer (5′ TGCAGAAAATTGATGCTGC-3′), 1 μl (10 pmole/ul) of the reverse primer (5′-TTGCCCAGGTTGGTAATAG-3′), 0.5 μl (10 pmole/ul) of the probe (FAM-ACCTGGGTGCGGTACAGAACCG-BHQ1), 8 μl of DW, and 12.5 μl of Premix EX Taq DNA polymerase (Takara, Japan) in 25 μl of total volume. PCR was carried out with an initial denaturation of 95°C for 10 min, followed by 40 cycles of 95°C for 10 sec and 64°C for 1 min. The CFU of *S. typhimurium* in each sample was calculated using a standard curve (Fig. 2).

### Histomorphological examination

The small intestinal tissues (jejunum) were fixed with 10% neutral buffered formalin. Subsequently, H&E staining for tissue samples was carried out as described previously [11]. The goblet cell density, VH, and crypt depth (CD) of each tissue were measured using the Diagnostic Insights visual analysis program (Olympus, Tokyo, Japan), as described previously [11]. Four parts (up, down, left, and right) of each H&E strained section were observed to measure VHs and CDs in 200× field, and the number of goblet cells in 400 × field, and then average values were calculated. 

### Statistical analysis

The experimental results were statistically analyzed using SPSS statistics 20 (IBM Corp., USA). First, all data were subjected to the normality test. Non-parametric data were analyzed by the Mann–Whitney test to compare two groups and the Kruskal–Wallis test for comparison of four groups, respectively. Parametric data were analyzed by independent sample *t*-test for comparison of two groups and Duncan’s test for comparison of four groups, respectively.

**Table 2.** Growth performance of post-weaning pigs challenged with *S.* *typhimurium*: effects of *S. typhimurium*-specific bacteriophage.

Experimental period		Non-challenged		Challenged	
		Group A	Group B	Group C	Group D
BW (kg)	Day 1	5.50 ± 0.40	5.45 ± 0.41	5.44 ± 0.45	5.56 ± 0.45
	Day 7	6.49 ± 0.69	6.52 ± 0.70	6.38 ± 0.37	6.66 ± 0.46
	Day 14	8.05 ± 0.56^a*^	8.18 ± 0.65^a^	7.02 ± 0.42^b^	7.67 ± 0.46^a^
ADG (gm)	Phase I (day 1–7)	142 ± 44	156 ± 42	134 ± 28	158 ± 11
	Phase II (day 7–14)	222 ± 23^a^	236 ± 29^a^	91 ± 24^c^	143 ± 23^b^

* Different letters (a–c) indicates significant difference (*p* < 0.05) between groups. Average daily feed intakes (ADI) of phase I and phase II were 277, 303, 270, and 298 gm, and 452, 461, 374, and 393 gm in Group A, B, C, and D respectively.

## Results

### Growth performance

In phase I (day 1–7), there was no significant difference in the BW and average daily gain (ADG) between the experimental groups. In phase II (day 7–14), the BW and ADG of Group C were significantly lower (*p* < 0.05) than that of Group D. These results indicate that the phage supplementation improved the impaired growth performance caused by *S. Typhimurium* infection (Table 2).

### Clinical signs

**Figure 3.**
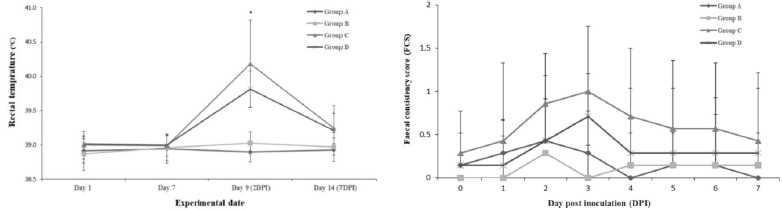
Changes in rectal temperature (left) and FCS (right) of post-weaning pigs during the experimental period: effects of *S. typhimurium*-specific bacteriophage. ^*^The challenged groups (Groups C and D) exhibited transiently the elevated temperature (*p* < 0.05) than non-challenged groups (Groups A and B) on 2 DPI.

The non-challenged groups maintained a steady level of rectal temperature during the experimental period, whereas the challenged groups showed transient fever (*p* < 0.05) on 2 DPI (day 9). The mean rectal temperatures were 38.9 ± 0.2, 39.0 ± 0.2, 40.2 ± 0.6, and 39.8 ± 0.3 in Groups A, B, C, and D, respectively (Fig. 3, left panel).

The fecal consistency score (FCS) did not change after the challenge of *S. typhimurium* in the non-challenged groups. FCS of the challenged group peaked on 3 DPI (day 10) and then decreased gradually. Group D exhibited lower FCS than Group C, although there was no significant difference between the groups (Fig. 3, right panel).

### Quantification of S. typhimurium in feces and small intestines

In non-challenged groups, *S. typhimurium* was not detected from feces and small intestinal tissues. The challenged groups excreted *S. typhimurium* via feces from 1 DPI (day 8). The level of excreted antigen was steadily maintained until 3 DPI (day 10) and then decreased on 7 DPI (day 14) in Group C. On the contrary, the level of excreted antigen was gradually reduced from 1 DPI to 7 DPI in Group D. Group D showed a lower quantificational value of *S. typhimurium* (*p* < 0.05) compared to Group C in the feces as well as the small intestinal tissues (Table 3). These results indicated that dietary phage supplementation reduced *S. typhimurium* colonization in the infected piglets*.*

**Table 3.** Amount of *S.* *typhimurium* in feces and small intestines: effects of *S. typhimurium*-specific bacteriophage.

Item		Non-challenged		Challenged	
		Group A	Group B	Group C	Group D
Fecal shedding of *S. typhimurium*	0 DPI (day 7)	-	-	-	-
	1 DPI (day 8)	-	-	5.8 ± 0.6^*^	3.5 ± 1.7 ^§^
	3 DPI (day 10)	-	-	5.7 ± 0.6	2.2 ± 2.1 ^§^
	7 DPI (day 14)	-	-	1.2 ± 2.0	0.3 ± 0.9
Small intestine (jejunum)		-	-	5.1 ± 0.9	2.8 ± 1.3 ^§^

* Quantificational value (log CFU/ml) in the samples by real-time PCR. ^§^Group D exhibited lower quantificational value (*p* < 0.05) than Group D.

### Morphology and goblet cell density of the intestinal tract

**Figure 4.**
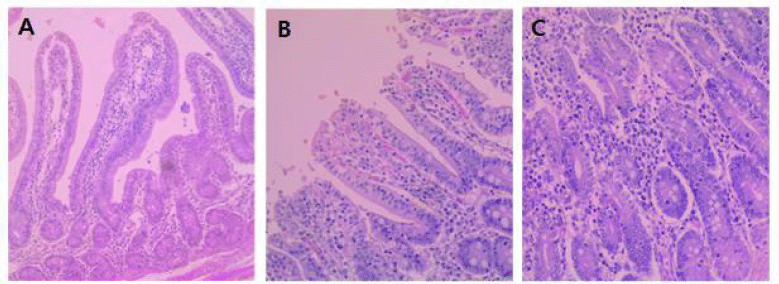
Histopathological changes of the intestinal tissues in post-weaning pigs challenged with *S. typhimurium.* (A) Normal intestinal morphology was sustained in the jejunum tissue section (H&E, 100×) from the non-challenged piglets in Group B. (B) Sever villous atrophy was observed in the jejunum tissue section (H&E, 200×) from the challenged piglets in Group C. (C) Crypt hyperplasia with lymphocyte infiltration was observed in the jejunum tissue section (H&E, 200×) from the challenged piglets in Group C.

In non-challenged groups (Groups A and B), there were no changes in the morphology of small intestines in response to dietary phage supplementation. In challenged groups (Group C and D), moderate to severe villous atrophy and crypt hyperplasia with lymphocyte infiltration was observed (Fig. 4). As a result, the decreased VH:CD ratio and goblet cell density were caused by the challenge of *S. typhimurium*. In comparison between Group C and D, there were significant changes in response to dietary phage supplementation. Group D showed a longer VH (*p* < 0.05) compared to Group C, and as a result, showed a higher VH:CD ratio (*p* < 0.05). Also, Group C exhibited a decreased number of goblet cells in the villi of small intestines compared to Group D (*p* < 0.05, Table 4).

## Discussion

In the livestock industry, large-scale intensive farming systems have been well established to meet the significant demands, such as for meat and milk. However, such production systems can easily promote bacterial disease transmission due to low genetic diversity and high stocking density, leading to simultaneous production and economic losses [12]. In this situation, antibiotic abuse is rampant in controlling such bacterial diseases. Accordingly, bacteria with resistance to several antibiotics are consistently reported worldwide. The emergence of antibiotic-resistant bacteria has become a significant problem in the livestock industry and human health. Thus, antibiotic alternatives are required to address this problem [13]. This study evaluated the protective effect of dietary supplementation with bacteriophage, which is one of the antibiotic alternatives, against *S. typhimurium* infection in post-weaning piglets.

The major manifestations of *S. typhimurium* infection are fever and yellowish watery diarrhea in weaning piglets [9]. In this study, the dietary supplementation of phage alleviates the clinical symptoms in the challenged piglets, although there were no significant differences in the rectal temperature and fecal consistency. After the challenge of *S. typhimurium*, the challenged piglets exhibited a transient increase in rectal temperature on 2 DPI, which were then recovered to the normal range. This result is similar to previous studies which reported that the rectal temperature of piglets tended to peak on 1–2 DPI and then decreased in the experimental inoculation of *S. typhimurium* [14,15].

**Table 4.** Effects of dietary supplementation of bacteriophage on morphology and goblet cell density of the intestinal tract in post-weaning pigs challenged with *S. typhimurium.*

Small intestine (Jejunum)	Non-challenged		Challenged	
	Group A	Group B	Group C	Group D
Morphology				
VH (μm)	369.9 ± 19.9^a§^	378.7 ± 21.3^a^	266.3 ± 24.1^c^	324.6 ± 18.0^b^
CD (μm)	238.1 ± 18.1^b^	255.6 ± 18.9^b^	329.2 ± 42.8^a^	305.6 ± 30.3^a^
VH:CD ratio^*^	1.56 ± 0.15^a^	1.48 ± 0.08^a^	0.82 ± 0.09^c^	1.07 ± 0.12^b^
Goblet cell density				
Villi	18.9 ± 1.7^a^	18.4 ± 1.2^a^	10.0 ± 1.8^b^	15.0 ± 4.8^a^
Crypt	38.0 ± 2.4^a^	36.7 ± 2.6^a^	16.0 ± 3.7^b^	21.1 ± 5.4^b^

^*^ VH/CD ratio. ^§^ Different letters (a–c) indicate significant differences (*p* < 0.05) between groups.

The dietary supplementation of phage had no impairment on growth performance in the non-infected piglets during phase I. On the contrary, it improved the growth performance in the piglets infected with *S. typhimurium*. This improvement is thought to be attributed to the reduction in *S. typhimurium* colonization by the phage. Also, the effect of the phage supplementation was supported by other factors such as bacterial shedding, the concentration of antigen in the small intestine, intestinal morphology, and goblet cell density in this study. Group D exhibited a lower quantificational value (log_10_ CFU/ml) of antigen in feces and small intestine tissues than Group D after the challenge.

VH and CD are indicators for intestinal absorptive capacity and morphological integrity, and the VH:CD ratio is regarded as a better indicator than either of the two metrics [11,16,17]. When pathogenic bacteria infect the intestinal epithelium, villous atrophy is induced by the inflammation caused by bacterial attachment and growth. Along with this, *crypt hyperplasia* due to increased stem cell proliferation in the crypt is promoted for the regeneration of intestinal enterocytes. These changes in intestinal morphology inhibit growth due to the reduction in nutrient absorption and intestinal brush-border enzymes activities [11,16,17]. In comparison to Group D, Group C had significantly lower VH and VH:CD ratios and greater CD. The goblet cells release mucin, a mucous material that acts as a gastrointestinal barrier, preventing pathogenic microorganisms from attaching to their enterocyte receptors before colonization [18]. Pathogenic bacteria infection in the intestinal tract causes a decrease in the goblet cells of intestinal mucosa [11,19]. Group C exhibited significantly decreased goblet cell density in villus and crypt compared to Group D. These results indicated that the phage supplementation effectively prevented the damage of the absorptive structures induced by *S. typhimurium* infection.

So far, some *in-vivo* experiments evaluating bacteriophage applications for swine salmonellosis caused by *S. typhimurium* have been conducted [4,5,8]. Most of these previous studies have focused on whether bacteriophage administration can reduce bacterial colonization in the infected pigs but did not investigate how much this reduction in bacterial colonization can affect growth performance [20–22]. However, this study confirmed that the dietary supplementation of bacteriophage could protect intestine integrity from *S. typhimurium* infection and ultimately improve growth rate.

Concerns have been raised about bacteriophage stability in acidic settings such as the stomach when bacteriophage therapy is administered orally. A few solutions, such as encapsulation or treatment with a buffering chemical, have been proposed to address this constraint [4,7]. The regimen supplemented with 1.0 × 10^9^ PFU/kg of STP-1 used in this study allowed reducing the bacterial colonization; as a result of efficiency, it improved the growth performance in the infected piglets. These results indicated that dietary supplement with this bacteriophage concentration is enough to deliver sufficient bacteriophage to the small intestine in *in-vivo* conditions without any techniques for protecting bacteriophage. If the bacteriophage protection techniques are excluded in the production process, it may facilitate benefit by reduced production cost in the economic aspect.

## Conclusion

If the phage protection technology is excluded from the production process, production costs will be reduced. The phage protection technology will reduce production costs if excluded from the production process. Besides *Salmonella*, there are other causative bacteria and there are other *Salmonella* organisms.

Collectively, the dietary supplementation of bacteriophage STP-1 (1.0 × 10^9^ PFU/kg) efficiently reduced *S. typhimurium* colonization in the small intestine. Through this, the intestinal integrity was protected, and the diarrhea symptoms were alleviated. Ultimately, the improved growth performance, which is the final goal of controlling post-weaning diarrhea [23], was confirmed in the infected piglets. This indicated that bacteriophage STP-1 would be economical and effective as a safe alternative to antibiotics for *S. typhimurium* infection in swine farms.

## List of Abbreviations

TSB, tryptic soy broth; CFU, colony-forming unit; PCR, polymerase chain reaction; IACUC, Institutional Animal Care and Use Committee; PFU, plaque-forming unit; BW, body weight; ADG, average daily gain; DPI, days post-inoculation; FCS, fecal consistency score; VH, villous height; CD, crypt depth; PWD, post-weaning diarrhea.
